# Calcitriol Prevents Cardiovascular Repercussions in Puromycin Aminonucleoside-Induced Nephrotic Syndrome

**DOI:** 10.1155/2018/3609645

**Published:** 2018-01-23

**Authors:** Fernandes-Cerqueira Cátia, Quelhas-Santos Janete, Sampaio-Maia Benedita, Simões-Silva Liliana, Soares-Silva Isabel, Roncon-Albuquerque Roberto, Pestana Manuel

**Affiliations:** ^1^Rheumatology Unit, Department of Medicine, Karolinska Institutet, Karolinska University Hospital Solna, CMM L8:04, 171 76 Stockholm, Sweden; ^2^Instituto Nacional de Engenharia Biomédica (INEB-I3S), Nephrology and Infectious Diseases Research and Development Group, University of Porto, Alameda Prof. Hernâni Monteiro, 4200-319 Porto, Portugal; ^3^Faculty of Dental Medicine, University of Porto, Rua Dr. Manuel Pereira da Silva, 4200-392 Porto, Portugal; ^4^Department of Physiology and Cardiothoracic Surgery General Practice, Faculty of Medicine, University of Porto, Al. Prof. Hernâni Monteiro, 4200-319 Porto, Portugal; ^5^Department of Nephrology, São João Hospital Center, EPE, Alameda Prof. Hernâni Monteiro, 4200-319 Porto, Portugal; ^6^Department of Renal, Urological and Infectious Diseases, Faculty of Medicine, University of Porto, Alameda Prof. Hernâni Monteiro, 4200-319 Porto, Portugal

## Abstract

Puromycin aminonucleoside-induced nephrotic syndrome (PAN-NS) is characterized by cardiac remodeling and increased local inflammatory activity. Patients with NS and animal models of NS have vitamin D3 deficiency. The aim of the present study was to evaluate the influence of calcitriol on cardiac remodeling and local inflammatory state in PAN-NS rat model. Male Sprague-Dawley rats were injected with PAN or vehicle on day 0. PAN and control rats were divided into two subgroups for the administration of calcitriol (PAN-D and Ct-D groups) or the vehicle (PAN-V and Ct-V groups) during 21 days. On day 21, the renal function, metabolic balance, calcitriol and FGF-23 plasma levels, prohypertrophy and proinflammatory markers (ET-1, TGF-*β*1, TNF-*α*, and IL-1*β*), and calcium signaling molecules (PLB and SERCA-2a) were evaluated. Twenty-one days after injection, PAN-V group presented cardiac hypertrophy and a modulation of proinflammatory markers local expression. Calcitriol treatment of PAN rats prevented cardiac hypertrophy and was associated with marked reduction in the cardiac expression levels of proinflammatory markers. Our results suggest that vitamin D3 deficiency in PAN-NS may contribute to cardiac remodeling and to the increase in local inflammatory activity. Calcitriol treatment prevents both cardiac repercussions and local inflammatory processes in PAN-NS.

## 1. Introduction

The nephrotic syndrome (NS) is characterized by increased proteinuria, accompanied by hypoalbuminemia, hyperlipidemia, lipiduria, and sodium retention that can lead to edema formation and ascites accumulation [1]. Additionally, the NS is associated with high incidence of heart diseases [2, 3]. Although dyslipidemia and hypercoagulability have been considered main factors implicated in heart disease in patients with NS [2, 3], other studies suggest that these classic cardiovascular risk factors cannot account alone for the high cardiovascular risk and mortality associated with proteinuria [4, 5].

Previous studies from our group showed that increased proteinuria in puromycin aminonucleoside-induced-NS (PAN-NS) rat model is accompanied by cardiac remodeling, impaired left ventricle function, and elevated cardiac proinflammatory activity [6]. These results suggest that protein wasting together with enhanced proinflammatory state may contribute to an increased cardiovascular risk in patients with NS.

Proteinuria due to glomerular injury and permeability dysfunction in NS has been associated with additional complications that result from the loss of vitamin D3 and its metabolites [1]. Plasma levels of vitamin D3 and metabolites are also reduced in patients with NS and normal renal function [7–9], as well as in animal models of NS [10, 11]. Although these studies provide evidence for a dysfunctional vitamin D3 metabolism in NS, the implications of calcitriol deficiency in this condition, namely, in the increased cardiovascular risk of these patients, remain to be elucidated.

Vitamin D3 is a steroid hormone that has a key function in the regulation of calcium, phosphate, and bone metabolism [1]. The active metabolite of vitamin D3, calcitriol, was suggested to have important beneficial effects in the cardiovascular system [12, 13]. Vitamin D3 therapy was described to contribute to increased survival among patients with chronic kidney disease [12, 14, 15]. Vitamin D receptor (VDR) knockout mice and vitamin D3-deficient rats were shown to have increased myocardial contractility along with cardiomyocytes hypertrophy and myocardial collagen accumulation [16].

On the basis of the previous considerations, the aim of the present study was to evaluate the effects of calcitriol supplementation on cardiac remodeling and local inflammatory activation in PAN-NS. Specifically, we evaluated the role of calcitriol in cardiac morphology, in the levels of proinflammatory and prohypertrophy markers (Endothelin-1 (ET-1), transforming growth factor-*β*1 (TGF-*β*1), tumor necrosis factor-*α* (TNF-*α*), and interleukin-1*β* (IL-1*β*)) and calcium signaling molecules (Phospholamban (PLB) and sarco/endoplasmic reticulum Ca^2+^-ATPase (SERCA-2a)). In addition, we studied the effect of calcitriol in the renal and metabolic function of PAN-NS animal model.

## 2. Materials and Methods

### 2.1. *In Vivo* Studies

All* in vivo* investigations were performed in accordance with the European Directive number 86/609, transposed to the Portuguese Law by DL 129/92 and by Portaria 1005/92.

#### 2.1.1. PAN-Induced Nephrosis

Normotensive male Sprague-Dawley rats (Harlan Laboratories Inc., Barcelona, Spain), weighing ~150 g, were selected after a seven-day period of stabilization. The animals received a single intraperitoneal injection (ip) of 10 mL/kg body weight (bw) of PAN (Sigma, St. Louis, MO, USA), 150 mg/kg bw (PAN group), or the vehicle, NaCl 0.9% (control (Ct) group), on day 0.

#### 2.1.2. Calcitriol Treatment

PAN and Ct groups were divided into two subgroups for the administration of calcitriol (Sigma), 50 ng/kg bw, ip (PAN-D, *n* = 4; Ct-D, *n* = 4), or the vehicle, propylene glycol diluted in 0.1% ethanol (v/v), ip (PAN-V, *n* = 3; Ct-V, *n* = 3). Calcitriol or vehicle was given daily from day 0 to day 20.

#### 2.1.3. Metabolic Studies and Tissue Collection

The animals were kept under controlled environmental conditions (12 : 12 hours' light/dark cycle and room temperature 22 ± 2°C). Four days before day zero, the rats were housed in metabolic cages (Tecniplast, Buguggiate (VA), Italy) for urine collection. In order to achieve the same daily sodium intake among the groups, the animals were fed as previously described [17]. Twenty-four-hour urine was collected in empty vials to measure proteins, calcium, phosphate, creatinine, sodium, and potassium levels. Animals were sacrificed on day 21 after PAN or vehicle injection. Blood was collected from the heart in vials containing lithium/heparin, centrifuged at 3800*g* for 15 minutes, and plasma was stored at −80°C for later determination of calcium, phosphate, creatinine, sodium, potassium, proteins, alkaline phosphatase (ALP), calcitriol, and fibroblast growth factor 23 (FGF-23). The heart was collected, weighed, and dissected: the right and the left ventricles together with the interventricular septum were separately weighed. The left ventricle (LV) was divided into several portions, some were kept in formalin for histological procedures, and others were frozen at −80°C for western-blotting and real time reverse transcription polymerase chain reaction (RT-PCR). Tibia length was measured for heart weight normalization.

### 2.2. *In Vitro* Studies

#### 2.2.1. Plasma and Urine Ionogram and Biochemistry

The quantification of sodium, potassium, total proteins, creatinine, and ALP was assayed in a Cobas Mira Plus analyzer (ABX Diagnostics, Switzerland) and the creatinine clearance, fractional excretion of sodium (FE_Na_^+^), and sodium balance were calculated as previously reported [18].

#### 2.2.2. Calcitriol and FGF-23 Determination

Calcitriol and FGF-23 levels in plasma samples were measured using commercial enzyme-linked immunosorbent assay kits following the manufacturer's protocol (Immundiagnostik, Bensheim, Germany, and Merck Millipore, Billerica, MA, USA, respectively).

#### 2.2.3. Heart Histology

LV samples were fixed in formalin, sectioned in 4 *μ*m thick slices (Electronic Rotary Microtome HM 340E, Microm, Thermo Fisher Scientific, Waltham, MA, USA), and stained with haematoxylin and eosin (H&E staining) for light microscope visualization (DM4000B, Leica Microsystems, Wetzlar, Germany). For myocardial hypertrophy assessment, the transversal diameter of cardiomyocytes was double-blindly measured in 70 cells randomly selected from eight to ten microscopic fields (Leica Application Suite Software, Leica Microsystems). To assess fibrosis, Sirius red staining was performed according to standard protocol.

#### 2.2.4. Immunohistochemistry

Left ventricle paraffin's 4 *μ*m thick slices were deparaffinized, hydrated in alcohol, and blocked with 3% H_2_O_2_ in methanol for ten minutes followed by incubation with goat anti-rabbit serum (Vectastain ABC kit, Vector Laboratories, Burlingame, CA, USA) for one hour at room temperature. Thereafter, slices were incubated with primary antibodies overnight at 4°C: rabbit polyclonal anti-TGF-*β*1 (1/50, Santa Cruz Biotechnology, Inc., Dallas, Texas, USA) and rabbit polyclonal anti-ET-1a (1/100, Santa Cruz Biotechnology Inc.). Immunostaining was detected using an anti-rabbit secondary biotinylated antibody (Diluted 1/100, Vector Laboratories) combined with an avidin-biotin complex (Vectastain ABC kit, Vector Laboratories), followed by 97% 3,3′-diaminobenzidine tetrahydrochloride hydrate (Sigma) and hematoxylin counterstaining.

#### 2.2.5. Western-Blotting

Primary antibodies used for protein detection were the following: mouse polyclonal anti-*β*-actin (1/20,000 Santa Cruz Biotechnology, Inc.), mouse monoclonal anti-TNF-*α* and anti-IL-1*β* (1/50, R&D Systems, 614 McKinley Place NE, MN, USA), mouse polyclonal anti-PLB (1/200, Affinity Bioreagents, Thermo Fisher Scientific), and rabbit polyclonal anti-SERCA-2a (1/1000, Cyclacel Pharmaceuticals Inc. Berkeley, Heights, NJ, USA). Left ventricle samples were homogenized with lysis buffer supplemented with phosphatase and protease inhibitors (Pierce Biotechnology, Thermo Fisher Scientific) solubilized in sample buffer [19] and denatured at 95°C for five minutes. Total proteins (30 *μ*g per well) were separated by sodium dodecyl sulfate polyacrylamide gel electrophoresis (12% acrylamide) and transferred onto a nitrocellulose membrane (Bio-Rad Laboratories, Inc., Hercules, CA, USA). Membranes were blocked with 5% nonfat dry milk in phosphate buffered saline containing 0.1% Tween 20, incubated with the primary antibodies overnight at 4°C, and lastly incubated for one hour at room temperature with fluorescently labelled goat anti-mouse (1/30,000 IRDye700, Rockland Immunochemicals, Inc., Pottstown, PA, USA) or donkey anti-rabbit (1/20,000, IRDye800, Rockland Immunochemicals, Inc.). Primary antibody binding was assessed by scanning the membranes with the Odyssey Infrared Imaging System (LI-COR Biosciences, Lincoln, NE, USA) and the intensity values were normalized for *β*-actin and shown as percentage of mean density of control rats.

#### 2.2.6. Real Time RT-PCR

mRNA expression quantification of pre-pro-ET-1, TGF-*β*1, SERCA-2a, PLB, TNF-*α*, IL-1*β*, and glyceraldehyde-3-phosphate dehydrogenase (GAPDH) genes in left ventricle samples was performed by real time RT-PCR as previously reported [20].

### 2.3. Statistics

Results are means ± SEM of values for the indicated number of determinations and were compared by one-way ANOVA followed by Student's *t*-test for unpaired comparisons. *P* < 0.05 was assumed to denote a significant difference.

## 3. Results

### 3.1. Characterization of PAN Nephrosis

**Figure 1:**
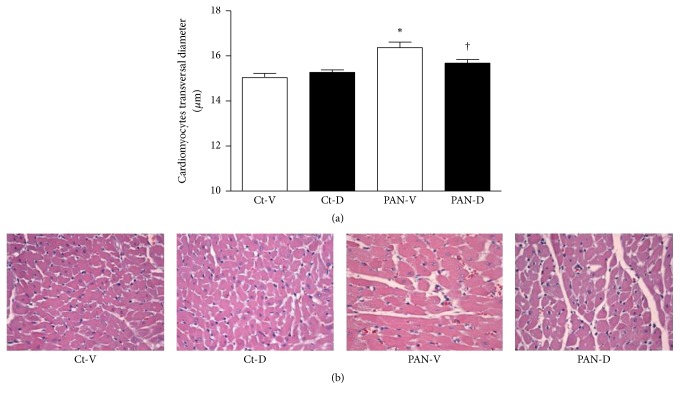
(a) Histomorphometry of the left ventricle in control (Ct-V and Ct-D) and nephrotic (PAN-V and PAN-D) rats, after 21 days of calcitriol (D) or vehicle (V) treatments. Cardiomyocytes cell size was determined by double-blind measure of the smallest transversal diameter. Data were obtained from 70 cells randomly selected from 8–10 microscopic fields. ^*∗*^*P* < 0.05 versus Ct-V rats; ^†^*P* < 0.05 versus PAN-V rats. (b) Light microscopy images of left ventricle cardiomyocytes from Ct-V, Ct-D, PAN-V, and PAN-D rats, 21 days after PAN or vehicle injection. Sections were stained with H&E (magnification 200x).

Renal function and other markers of metabolic balance are described in Table 1 for control (Ct-V and Ct-D) and nephrotic (PAN-V and PAN-D) rats, after 21 days of calcitriol or vehicle treatments. The protein excretion remained elevated in PAN rats up to 21 days after PAN injection (Figure 1). At this stage, the urinary Na^+^ excretion and the FE_Na_^+^ were similar among groups, indicating that the animals had reached a status of Na^+^ balance (Figure 1 and Table 1). Albeit all animals had the same food intake throughout the study, the body weight of PAN rats was significantly reduced in comparison to the corresponding control groups at the end of the study (Table 1).

Fractional excretion of Ca^2+^ (FECa2^+^) was reduced in PAN rats in comparison to the corresponding control group, although no statistical significance was observed (Table 1). Fractional excretion of P (FE P) and creatinine clearance were similar among the four groups (Table 1).

### 3.2. Cardiac Morphology and Histomorphometry

Twenty-one days after PAN injection, we observed a cardiac hypertrophy, evidenced by increased heart weight/tibial length ratio and LV/tibial length ratio (Table 2), as well as increased cardiomyocytes diameter (Figures 1(a) and 1(b)). Calcitriol treatment significantly reverted the increased cardiomyocytes diameter in PAN-treated group (Figure 1) but not the heart weight/tibial length ratio and the LV/tibial length ratio (Table 2).

### 3.3. Cardiac Calcium-Handling Molecules

**Figure 2:**
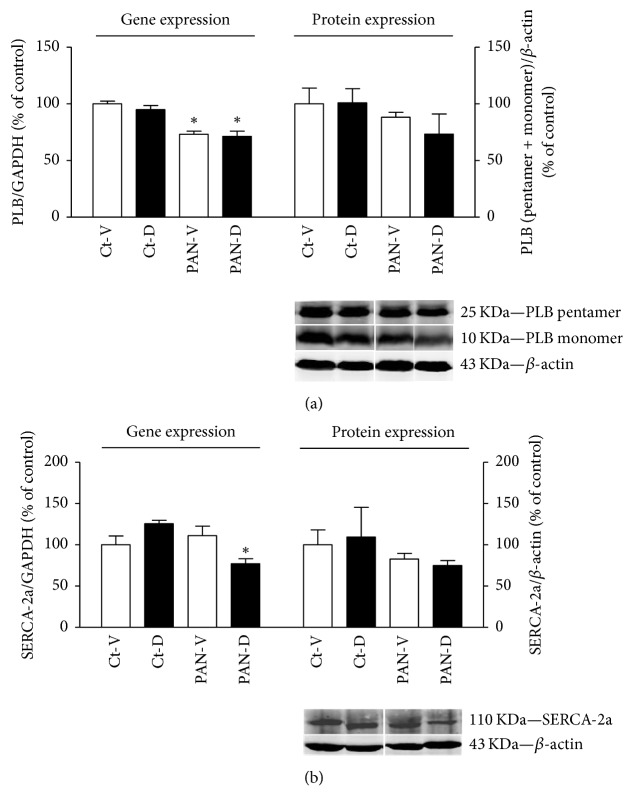
(a) Top: left ventricle mRNA and protein expression of total PLB in control (Ct-V and Ct-D) and nephrotic (PAN-V and PAN-D) rats, after 21 days of calcitriol (D) or vehicle (V) treatments. mRNA and protein expression were normalized for GAPDH gene and *β*-actin protein, respectively. Results are expressed as % of control. ^*∗*^*P* < 0.05 versus correspondent control rats. Bottom: representative immunoblots of PLB (pentamer 25 KDa and monomer 10 KDa bands) and *β*-actin (43 KDa band). (b) Top: left ventricle mRNA and protein expression of SERCA-2a in control (Ct-V and Ct-D) and nephrotic (PAN-V and PAN-D) rats, after 21 days of calcitriol (D) or vehicle (V) treatments. mRNA and protein expression were normalized for GAPDH gene and *β*-actin protein, respectively. Results are expressed as % of control. ^*∗*^*P* < 0.05, versus Ct-V group. Bottom: representative immunoblots of SERCA-2a and *β*-actin (110 KDa and 43 KDa bands, respectively).

PLB gene expression was significantly reduced by PAN injection but not influenced by calcitriol supplementation (Figure 2). A similar profile was observed in PLB protein expression, but without statistical significance.

Twenty-one days after PAN or vehicle injection, LV gene expression of PLB was significantly reduced in PAN-treated groups (PAN-V and PAN-D) in comparison to their corresponding controls (Ct-V and Ct-D) (Figure 2(a), left panel). However, no significant differences were observed in LV protein expression of PLB among the four groups (Figure 2(a), right panel).

PAN animals treated with calcitriol showed significantly reduced gene expression of SERCA-2a in LV in comparison to the control group Ct-D (Figure 2(b), left panel).

### 3.4. Cardiac Hypertrophy and Proinflammatory Activity

**Figure 3:**
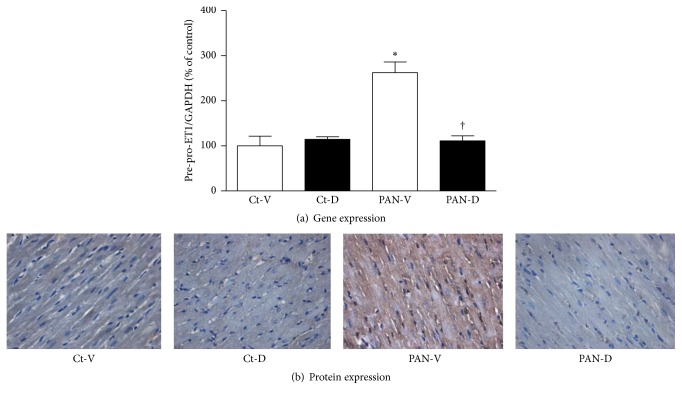
(a) Left ventricle mRNA expression of pre-pro-ET1 in control (Ct-V and Ct-D) and nephrotic (PAN-V and PAN-D) rats, after 21 days of calcitriol (D) or vehicle (V) treatments. mRNA expression was normalized for GAPDH gene and the results are expressed in % of control. ^*∗*^*P* < 0.05 versus Ct-V rats; ^†^*P* < 0.05 versus PAN-V rats. (b) Immunohistochemical detection of ET-1a in left ventricle of control (Ct-V and Ct-D) and nephrotic (PAN-V and PAN-D) rats, after 21 days of calcitriol (D) or vehicle (V) treatments (magnification 200x).

No significant difference was found in pre-pro-ET1 gene expression in LV between Ct-V and Ct-D groups (Figure 3(a)). Twenty-one days after PAN or vehicle injection, PAN-V group presented a 2.5-fold increase of pre-pro-ET1 gene expression in the LV in comparison with Ct-V group (Figure 3(a)). By contrast, PAN rats treated with calcitriol presented pre-pro-ET1 LV gene expression levels similar to those observed in the corresponding control group (Ct-D) (Figure 3(a)).  Figure 3(b) illustrates immunohistochemistry images of ET-1a in LV paraffin sections of the four groups of animals. A marked increase in the protein expression of ET-1a was observed in the LV of PAN-V group in comparison with both Ct-V and PAN-D groups.

**Figure 4:**
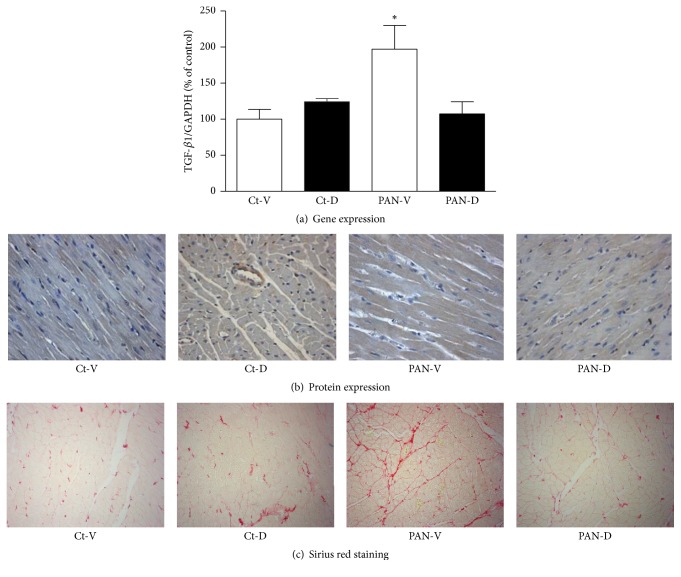
(a) Left ventricle mRNA expression of TGF-*β*1 in control (Ct-V and Ct-D) and nephrotic (PAN-V and PAN-D) rats, after 21 days of calcitriol (D) or vehicle (V) treatments. mRNA expression was normalized for GAPDH gene and the results are expressed in % of control. ^*∗*^*P* < 0.05, versus Ct-V rats. (b) Immunohistochemical detection of TGF-*β*1 in left ventricle of control (Ct-V and Ct-D) and nephrotic (PAN-V and PAN-D) rats, after 21 days of calcitriol (D) or vehicle (V) treatments (magnification 200x). (c) Sirius red staining illustrating fibrosis significantly increased in PAN-V rats in comparison to Ct-V and PAN-D rats, after 21 days of calcitriol (D) or vehicle (V) treatments (magnification 200x).

No significant difference was found in TGF-*β*1 gene expression in LV between Ct-V and Ct-D groups (Figure 4(a)). Twenty-one days after PAN or vehicle injection, PAN-V group presented a 2-fold increase of TGF-*β*1 gene expression in the LV in comparison with Ct-V group (Figure 4(a)). By contrast, PAN rats treated with calcitriol presented TGF-*β*1 LV gene expression levels similar to those observed in the corresponding control group (Ct-D) (Figure 4(a)).  Figure 4(b) illustrates immunohistochemistry images of TGF-*β*1 in LV paraffin sections of the four groups of animals. In agreement with previous results, a marked increase in protein expression of TGF-*β*1 was observed in the LV of PAN-V group in comparison with both Ct-V and PAN-D groups. Microscopy images of Sirius red staining were obtained to visualize cardiac fibrosis (red color denotes connective tissue staining). As can be observed in Figure 4(c), PAN-V rats presented stronger staining for connective tissue in the LV in comparison with vehicle-treated rats (Ct-V, Figure 4(c)). In PAN rats treated with calcitriol, the connective tissue staining was markedly attenuated in comparison to PAN-V group (Figure 4(c)).

**Figure 5:**
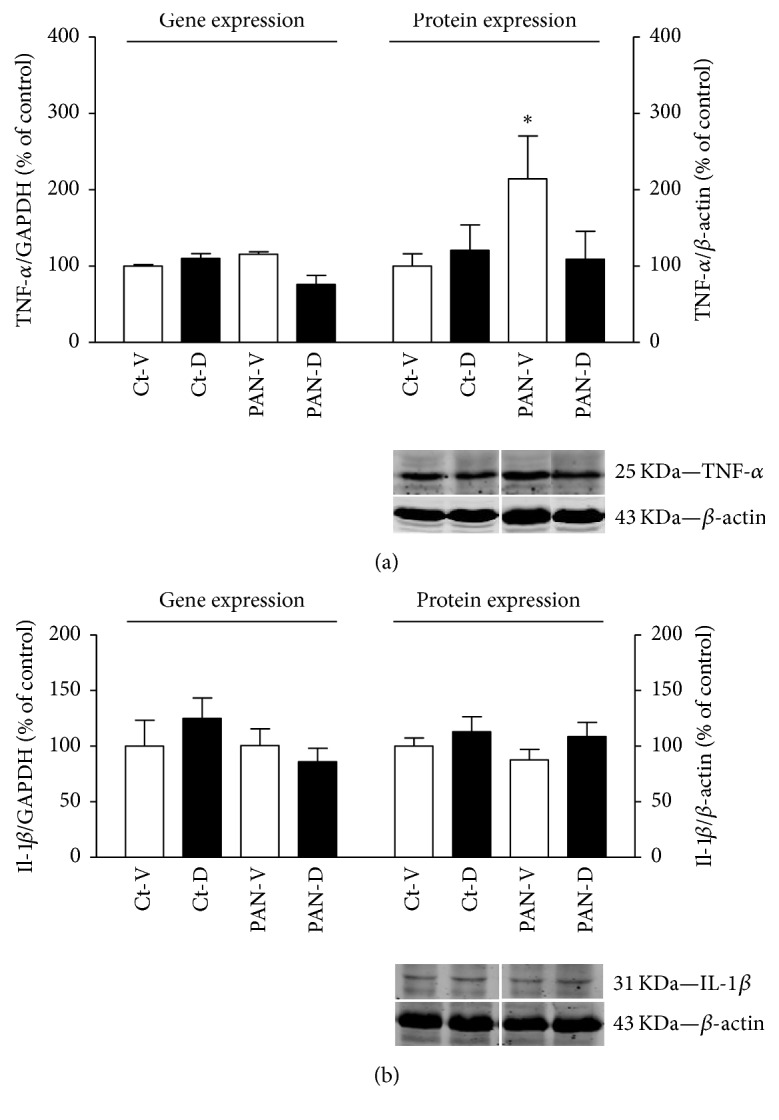
(a) Top: left ventricle mRNA and protein expression of TNF-*α* in control (Ct-V and Ct-D) and nephrotic (PAN-V and PAN-D) rats, after 21 days of calcitriol (D) or vehicle (V) treatments. mRNA and protein expression were normalized for GAPDH gene and *β*-actin protein, respectively. Results are expressed as % of control. ^*∗*^*P* < 0.05 versus Ct-V rats. Bottom: representative immunoblots of TNF-*α* and *β*-actin (25 KDa and 43 KDa bands, respectively). (b) Top: left ventricle mRNA and protein expression of IL-1*β* in control (Ct-V and Ct-D) and nephrotic (PAN-V and PAN-D) rats, after 21 days of calcitriol (D) or vehicle (V) treatments. mRNA and protein expression were normalized for GAPDH gene and *β*-actin protein, respectively. Results are expressed as % of control. Bottom: representative immunoblots of IL-1*β* and *β*-actin (31 KDa and 43 KDa bands, respectively).

TNF-*α* mRNA levels in the LV did not differ among the four groups (Figure 5(a), left panel). By contrast, TNF-*α* protein expression was elevated in PAN-V rats in comparison to Ct-V group (Figure 5(a), right panel). PAN rats treated with calcitriol (PAN-D) presented levels of TNF-*α* protein expression similar to those found in the corresponding control group (Ct-D) (Figure 5(a), right panel). No significant differences were observed in IL-1*β* protein and gene expression in the myocardial tissue, among the four groups (Figure 5(b)).

### 3.5. Calcitriol and FGF-23 Plasma Levels

**Figure 6:**
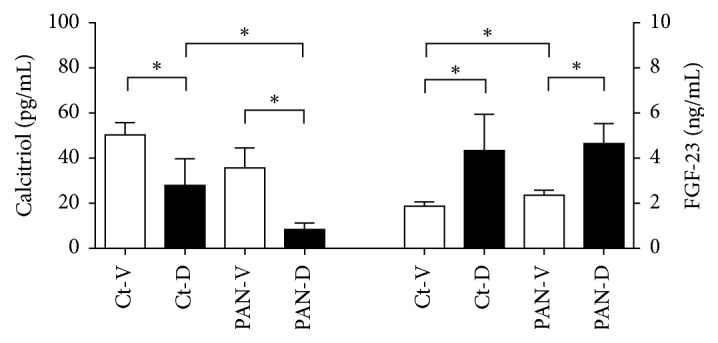
Calcitriol (left panel) and FGF-23 (right panel) plasma levels in control (Ct-V and Ct-D) and nephrotic (PAN-V and PAN-D) rats, after 21 days of calcitriol (D) or vehicle (V) treatments. ^*∗*^*P* < 0.05.

Calcitriol plasma levels were reduced in both Ct and PAN groups of rats treated with calcitriol (Figure 6, left panel). In addition, calcitriol plasma levels were significantly reduced in PAN rats treated with calcitriol in comparison with the corresponding control group (Ct-D). By contrast, FGF-23 plasma levels were significantly increased in calcitriol-treated rats in both Ct and PAN groups (Figure 6, right panel).

## 4. Discussion

In the present study, we evaluated the influence of calcitriol supplementation on cardiac remodeling and enhanced local inflammatory activity in PAN-NS rat model. Calcitriol supplementation in PAN-NS prevented cardiac hypertrophy, fibrosis, and inflammation, namely, by downregulating ET-1a, TGF-*β*1, and TNF-*α* genes and protein expression. Taken together, our results suggest that calcitriol supplementation has important beneficial effects in the myocardial dysfunction observed in NS.

Previous results from our group, obtained seven and fourteen days after PAN injection, demonstrated that cardiac remodeling and dysfunction are accompanied by cardiac inflammatory activation [20]. In the present study, we show that 21 days after PAN injection, the nephrotic animals maintain a dysregulated myocardial structure and function evidenced by cardiomyocytes hypertrophy, elevated fibrosis, and enhanced proinflammatory activity. The current study evaluated the effect of daily treatment with calcitriol for 21 days in cardiac dysfunction displayed by PAN-NS rats. Calcitriol supplementation to nephrotic animals during 21 days prevented the increase of both the ET-1a and TGF-*β*1 levels observed in the nephrotic control group. These findings together with the reduction of connective tissue staining observed in the left ventricle of PAN rats treated with calcitriol indicate a protective effect of calcitriol in cardiac hypertrophy and fibrosis.

Previous reports state that cardiac myocytes have a functional vitamin D3 system that plays an important role as an antihypertrophic agent [21, 22]. Calcitriol or its nonhypercalcemic analogues were suggested to antagonize ET-1a induced hypertrophy* in vitro* by modulating the expression of hypertrophy regulatory genes [23]. Additionally, spontaneously hypertensive heart failure rats that were fed a high-salt diet and treated with calcitriol presented lower heart weight and left ventricular diameter, thus suggesting that calcitriol behaves as an antihypertrophy factor [22]. Mesenchymal multipotent cells treated with calcitriol showed increased expression of VDR and antifibrotic factors and decreased expression of TGF-*β*1 and collagen types I and III [24]. Collectively these findings together with our results lead to the hypothesis that vitamin D3 deficiency in PAN-NS may contribute to cardiac dysfunction due to an exacerbated hypertrophic state.

The local production of proinflammatory factors such as TNF-*α* in the heart is potentially relevant due to the well-known effects on cardiac remodeling and contractile dysfunction [25, 26]. Furthermore, patients with focal segmental glomerular sclerosis presented higher TNF-*α* plasma and urine levels in comparison to those registered by healthy controls [27]. In line with this, peripheral blood mononuclear cells isolated from patients with primary nephrotic syndrome showed enlarged TNF-*α* production in comparison with controls [28]. Although the urinary or systemic levels of TNF-*α* were not assessed in this study, we observed a marked increase in the cardiac levels of TNF-*α* protein expression. Interestingly, the increased expression of TNF-*α* observed in the nephrotic animals was prevented by the administration of calcitriol. Our results are in accordance with a previous study whereby human monocytes stimulated by interferon or phorbol esters displayed an impaired production of TNF-*α* [29]. Although IL-1*β* was described to be a major proinflammatory mediator in children with nephrotic syndrome and in animal models of nephrosis [30–32], in the present study no variations were observed in IL-1*β* expression levels in PAN-treated rats compared to the control group. Likewise, calcitriol did not exert any action on this proinflammatory cytokine.

SERCA-2a is the main protein involved in calcium reuptake into the sarcoplasmic reticulum of cardiomyocytes and the modulation of its activity is dependent on PLB phosphorylation [33]. In our previous study [20], seven and fourteen days after PAN injection, nephrotic rats presented reduced cardiac protein levels of both SERCA-2a and PLB; however, at 21 days after PAN injection, no major alterations were observed in these protein levels. These findings suggest that, on a later stage of the animal model of PAN-NS, SERCA-2a and PLB may not be important contributors to myocardial dysfunction. Although vitamin D3 was shown to be involved in the contractile function of the heart by regulating calcium cycling in adult myocytes [34], herein calcitriol supplementation appears not to be a relevant factor in the regulation of PAN-NS-calcium signaling.

Patients with NS as well as nephrotic rats are known to have vitamin D3 deficiency due to loss in urine as a result of impaired renal function [7, 10]. In the present study, the supplementation with calcitriol was expected to induce a raise in calcitriol blood levels. However, after 21 days of calcitriol daily supplementation (at the end of the experiment), we unexpectedly observed a noteworthy reduction in the calcitriol blood levels in both control and nephrotic animals. Yadav et al. showed that FGF-23 is reduced in subjects with untreated nephrotic syndrome and suggested that the reduced levels of vitamin D and urinary losses may contribute to lower levels of FGF-23 in NS [35]. Interestingly, in our study, calcitriol treatment leads to a significant increase in FGF-23 plasma levels in both control and nephrotic animals. In addition, although the food intake was equivalent in all groups, calcitriol-treated animals presented slightly higher phosphaturia in comparison to vehicle-treated rats. The main physiological function of FGF-23 is to stimulate phosphaturia and thereby reduce calcitriol levels. In order to do that, FGF-23 acts by inhibiting renal 1*α*-hydroxylase and stimulating 24-hydroxylase [36]. Chronic kidney disease patients have reduced viable nephrons and elevated FGF-23 which impairs activation of 1*α*-hydroxylase and therefore leads to low serum levels of 25-hydroxy vitamin D and consequently calcitriol [36]. Our results show a rise in FGF-23 plasma levels in response to calcitriol supplementation in order to maintain phosphate homeostasis in calcitriol-treated rats.

In summary, our results show that calcitriol supplementation to PAN-NS rats prevents cardiovascular remodeling and enhanced left ventricle inflammatory activity, namely, by preventing cardiomyocytes hypertrophy and by decreasing gene and protein expression of several proinflammatory molecules. These findings suggest that vitamin D3 system may be endowed with an important protective role in cardiac remodeling and dysfunction in NS.

## Figures and Tables

**Table 1:** Body weight, metabolic balance, and renal function in control (Ct-V and Ct-D) and nephrotic (PAN-V and PAN-D) rats, after 21 days of calcitriol or vehicle treatments.

	Day 21			
	Ct-V	Ct-D	PAN-V	PAN-D
Body weight (g)	240 ± 3	237 ± 5	205 ± 6^*∗*^	183 ± 5^*∗*,†^
Food intake (g/24 h)	0.02 ± 0.00	0.02 ± 0.00	0.02 ± 0.00	0.02 ± 0.00

P proteins (g/L)	52.7 ± 3.4	51.4 ± 0.4	48.8 ± 2.6	53.0 ± 2.5
U proteins (mg/24 h)	15.3 ± 2.4	17.0 ± 2.2	358 ± 37^*∗*^	413 ± 53^*∗*^

Na^+^ intake (mmol/24 h)	1.65 ± 0.00	1.65 ± 0.00	1.65 ± 0.00	1.65 ± 0.00
P Na^+^ (mmol/L)	146 ± 5	149 ± 2	143 ± 3	150 ± 1
U Na^+^ (mmol/24 h)	1.31 ± 0.23	1.40 ± 0.05	1.38 ± 0.14	1.44 ± 0.09
Na^+^ balance (mmol/24 h)	0.35 ± 0.23	0.26 ± 0.05	0.27 ± 0.14	0.21 ± 0.09
FE Na^+^ (%)	0.34 ± 0.07	0.67 ± 0.23	0.57 ± 0.10	0.60 ± 0.16

P Ca^2+^ (mmol/L)	2.45 ± 0.15	3.13 ± 0.08^*∗*^	2.84 ± 0.47	3.03 ± 0.09
U Ca^2+^ (mmol/24 h)	0.26 ± 0.12	0.04 ± 0.02	0.01 ± 0.00	0.02 ± 0.01
FE Ca^2+^ (%)	4.57 ± 3.00	3.58 ± 1.77	0.17 ± 0.03	0.52 ± 0.20

P P (mmol/L)	2.96 ± 0.24	3.33 ± 0.39	3.10 ± 0.23	3.52 ± 0.16
U P (mmol/24 h)	0.06 ± 0.04	0.12 ± 0.03	0.08 ± 0.05	0.10 ± 0.03
FE P (%)	0.53 ± 0.23	1.37 ± 0.43	0.43 ± 0.21	1.27 ± 0.26

P creatinine (mg/L)	3.73 ± 0.29	5.07 ± 0.58	4.70 ± 0.59	3.27 ± 0.61
C creatinine (mL/min)	2.44 ± 0.74	1.76 ± 0.15	1.49 ± 0.27	1.75 ± 0.59
P ALP (U/I)	147 ± 23	141 ± 8	101 ± 21	98 ± 8^*∗*^

Values are means ± SE; *n* = 3 to 4 experiments per group. P, plasma; U, urine; FE, fractional excretion; and C, clearance. ^*∗*^*P* < 0.05, significantly different from values in the corresponding control groups (Ct-V or Ct-D); ^†^*P* < 0.05, significantly different from corresponding values in PAN-V group.

**Table 2:** Characterization of cardiac morphometry in control (Ct-V and Ct-D) and nephrotic (PAN-V and PAN-D) rats, after 21 days of calcitriol (D) or vehicle (V) treatments.

	Day 21			
	Ct-V	Ct-D	PAN-V	PAN-D
Heart weight (mg)	923.3 ± 5.0	940.3 ± 27.2	960.0 ± 8.9^*∗*^	933.8 ± 18.8
Heart weight/tibial length (mg/mm)	245.3 ± 4.8	249.9 ± 9.7	269.2 ± 1.8^*∗*^	262.9 ± 6.2
LV weight/tibial length (mg/mm)	173.6 ± 7.0	178.8 ± 7.0	198.7 ± 6.2^*∗*^	196.2 ± 3.2
RV weight/tibial length (mg/mm)	37.1 ± 1.7	40.2 ± 1.5	34.9 ± 1.5	31.6 ± 1.6

Values are means ± SE; *n* = 3 to 4 experiments per group; LV, left ventricle; RV, right ventricle; ^*∗*^*P* < 0.05, significantly different from corresponding values in Ct-V group.
